# The interaction of bile acids and gut inflammation influences the pathogenesis of inflammatory bowel disease

**DOI:** 10.1007/s11739-023-03343-3

**Published:** 2023-07-29

**Authors:** Agostino Di Ciaula, Leonilde Bonfrate, Mohamad Khalil, Piero Portincasa

**Affiliations:** Clinica Medica “A. Murri” and Division Internal Medicine, Department of Precision and Regenerative Medicine and Ionian Area (DiMePre-J), University “Aldo Moro” Medical School, Policlinico Hospital, Piazza G. Cesare 11, 70124 Bari, Italy

**Keywords:** Crohn’s disease, Enterohepatic circulation, FXR, GPBAR1, Gut barrier, Gut microbiota, Nuclear receptors, Ulcerative colitis

## Abstract

Bile acids (BA) are amphipathic molecules originating from cholesterol in the liver and from microbiota-driven biotransformation in the colon. In the gut, BA play a key role in fat digestion and absorption and act as potent signaling molecules on the nuclear farnesoid X receptor (FXR) and membrane-associated G protein-coupled BA receptor-1 (GPBAR-1). BA are, therefore, involved in the maintenance of gut barrier integrity, gene expression, metabolic homeostasis, and microbiota profile and function. Disturbed BA homeostasis can activate pro-inflammatory pathways in the gut, while inflammatory bowel diseases (IBD) can induce gut dysbiosis and qualitative and/or quantitative changes of the BA pool. These factors contribute to impaired repair capacity of the mucosal barrier, due to chronic inflammation. A better understanding of BA-dependent mechanisms paves the way to innovative therapeutic tools by administering hydrophilic BA and FXR agonists and manipulating gut microbiota with probiotics and prebiotics. We discuss the translational value of pathophysiological and therapeutic evidence linking BA homeostasis to gut inflammation in IBD.

## Introduction

Bile acids (BA) are amphipathic lipid components of the human bile with non-esterified cholesterol and phospholipids. The BA pool is composed of primary BA synthesized from cholesterol in the liver and secondary BA from microbiota-driven biotransformation in the colon. In the gut, BA are involved in the emulsification and absorption of dietary fat and fat-soluble vitamins [1], have regulatory functions on epithelial cell proliferation [2–4] and gut barrier [4], affect expression of several genes involved in metabolic homeostasis [1, 5–7], stimulate epigenetic profiles [8, 9], modulate gut microbiota [6, 10], and have antimicrobial properties [10]. Substantial changes of the BA pool also affect the integrity of the intestinal barrier [4, 11], promote immune-modulatory effects [12–14], and modulate inflammatory pathways through signaling mechanisms that involve the nuclear receptor farnesoid X receptor (FXR) [15] and the membrane-associated G-protein-coupled BA receptor-1 (GPBAR1).

Evidence points to a close link between BA homeostasis and gut integrity in health and disease. Inflammatory bowel disease (IBD) is associated with disturbances in the gut microbiota and immune imbalance, which, in parallel with the influence of environmental factors, can greatly affect the integrity of the gut barrier [16]. In addition, IBD patients display a consistent shift of the BA pool, e.g., increased fecal concentrations of primary and conjugated BA [17].

In this review, we discuss the bidirectional intersection of BA homeostasis and chronic intestinal inflammation considering novel therapeutic approaches. In recent reviews, we focused on specific aspects of BA homeostasis, enterohepatic circulation, and function as signaling molecules [7, 15].

## BA synthesis secretion and absorption

Primary BA (cholic acid [CA] and chenodeoxycholic acid [CDCA]) are synthetized as catabolic products of cholesterol in the pericentral hepatocyte and undergo subsequent conjugation with taurine (2-aminoethanesulfonic acid) and the amino acid glycine (ratio 3:1) through N-acyl amidation at carbon 24 of the aliphatic side chain [18]. This step increases BA solubility in bile (an aqueous solutions) and decreases BA toxicity. BA are actively secreted mainly by the bile salt export pump (BSEP; ABCB11/Abcb11) into the canaliculi [19] and then appear in bile, stored and concentrated in the gallbladder and periodically delivered to the intestine during fasting and mainly during the fat-cholecystokinin-dependent stimulation of the gallbladder in the postprandial period [1].

Reabsorption of about 95% of BA occurs in terminal ileum with uptake by the apical sodium-dependent bile salt transporter (ASBT; SLC10A2/Slc10a2) [20] and binding and transport across the enterocyte by the ileal BA-binding protein (IBABP) [21, 22]. The basolateral BA efflux into the portal circulation requires a third transporter, the organic solute transporters (OSTα and OSTβ heterodimer) [23]. The hepatic reuptake of BA occurs at the basolateral (sinusoidal) membrane, and requires the sodium taurocholate co-transporting polypeptide (NTCP; SLC10A1/Slc10a1) [24]. The sodium-independent basolateral BA uptake into hepatocytes accounts for only 25% of the uptake of mainly unconjugated BA, and is mediated by organic anion transporting polypeptides (OATPs) [1, 19, 25]

**Fig. 1:**
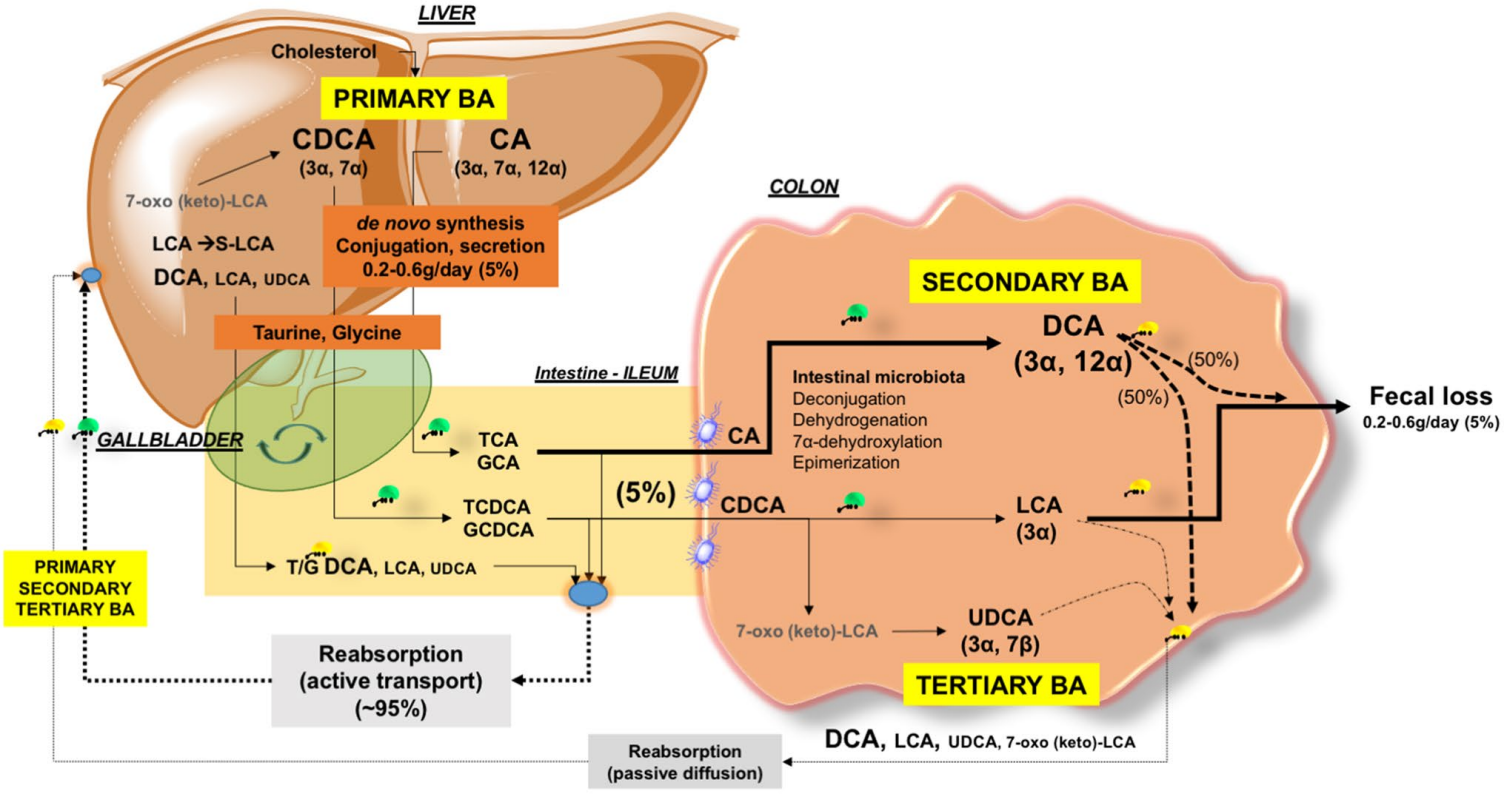
The enterohepatic circulation of bile acids (BA) and qualitative/quantitative composition of the BA pool. Events linked to the synthesis, conjugation, secretion, biotransformation, re-absorption, and excretion of primary, secondary, and tertiary bile acids (BA) in humans at every cycle of the enterohepatic circulation. “Primary” BA, synthetized in the liver starting from cholesterol are the trihydroxy cholic acid (CA) hydroxylated at the 3α,7α,12α positions and the dihydroxy chenodeoxycholic acid (CDCA) hydroxylated at the 3α,7α positions. The two biosynthetic pathways are the classical pathway involving the 7a-hydroxylase which stimulates the 7a-hydroxylation of cholesterol. This major enzyme contributes to more than 75% of total production of primary BA. The alternative pathway is initiated by the sterol-27-hydroxylase which produces mainly CA. BA are actively re-absorbed at the terminal ileum. About 5% of primary BA escape ileal absorption and enter the colon where the resident microbiota initiate BA deconjugation from taurine and glycine, dehydrogenation, dehydroxylation, and epimerization to produce «secondary» BA: the dihydroxy deoxycholic acid (DCA) hydroxylated at the 3α,12α positions and the monohydroxy lithocholic acid (LCA) hydroxylated at the 3α position. The 7α-dehydrogenation of CDCA form the dihydroxy 7α-oxo (keto)-LCA which is metabolized to the “tertiary’ 7β-epimer, the dihydroxy hydrophilic ursodeoxycholic acid (UDCA) hydroxylated at the 3α,7β positions in the colon and to CDCA again in the liver. The 7α-dehydroxylation of the primary BA is the essential reaction to produce DCA and LCA from CA and CDCA, respectively. Both LCA, 7-oxo (keto)-LCA, and UDCA are mainly excreted in feces while about 50% DCA is passively reabsorbed from the colon into the portal tract [27] by ionic more than nonionic diffusion (the remaining part being excreted with feces). Their fate depends on specific physicochemical properties and BA binding to luminal contents. In the liver, a small amount of LCA is quickly transformed in the sulphonated “tertiary” (S-LCA). Altogether, the BA pool at every cycle undergoes re-conjugation with taurine and glycine and new secretion in bile. Fecal loss is minimal (< 5% at every cycle). As an example, when a CA or CDCA pool of 1 g cycles 6 times a day, the daily loss is 5% × 6 cycles = 30% and 300 mg must be resynthesized in the liver [25]. **B** Relative composition of hepatic and gallbladder bile in health as main solutes (left) and individual bile acids (right). Altogether, the glyco-, tauro-conjugated CA, CDCA and DCA represent more than 90% of the total pool of BA. Abbreviations: *G/T* glycine, taurine, *CA* cholic acid, *CDCA* chenodeoxycholic acid, *DCA* deoxycholic acid, *LCA* lithocholic acid, *UDCA* ursodeoxycholic acid

A small amount of primary BA escapes ileal re-absorption and enters the colon, where the resident microbiota promotes the deconjugation, dehydrogenation, and dihydroxylation of primary BA to secondary BA, mainly deoxycholic acid (DCA), small amount of lithocholic acid (LCA), and the “tertiary” ursodeoxycholic acid (UDCA). This additional pool of colonic unconjugated BA undergoes passive diffusion, i.e., ~ 50% DCA, minimal LCA (both mainly insoluble) and UDCA and is transported back to the liver through the portal circulation where both secondary and tertiary BA are conjugated again with taurine or glycine in the liver and re-secreted [26]. This amount of colonic BA which is passively reabsorbed contributes to the enterohepatic circulation of BA with 95% re-absorption at every cycle [27]. The remaining DCA, and a small amount of LCA and UDCA are lost in the feces, accounting for ~ 5% of the total BA pool at every cycle [28]. In health, this BA fecal loss is a fraction of the total amount lost daily according to the number of enterohepatic cycles, and must be compensated by the daily de novo synthesis in the liver [29, 30] (Fig. 1). In general, if the pool cycles 2–3 times per meal, according to the meal frequency, size, and composition, i.e., 4–12 times/day, this increases the BA pool to a “dynamic” size (3 g x − 12 cycles = 12–36 g/day), and a capacity to reabsorb 10–30 g of BA per day [1].

## Deranged BA homeostasis

IBD patients can develop changes of BA synthesis and enterohepatic circulation, both predisposing factors to deranged BA homeostasis. Table 1 depicts the main pathogenic mechanisms able to derange BA homeostasis in IBD patients [17]. In general, mechanisms include changes of BA absorption, microbiota biosynthesis, altered signaling mechanisms, and or deranged BA metabolism.

**Table 1:** Putative mechanisms deranging bile acid (BA) homeostasis in inflammatory bowel diseases

Change	Consequence
Decreased expression and/or function of the apical sodium-dependent bile acid transporter (ASBT)	Altered re-absorption of conjugated BA in the terminal ileum [204–209]
Surgical resection of the distal ileum	Decreased re-absorption of conjugated BA in the terminal ileum [209]
BA malabsorption, intestinal inflammation [210], genetic factors [211]	Decreased FXR activation, decreased inhibition of hepatic BA synthesis, increased BA production [36, 66]
Altered GPBAR1 gene expression [74] and subsequent	Altered modulation of BA synthesis, intestinal biotransformation, and uptake [7]
Gut dysbiosis	Deranged BA biotransformation [34, 86, 212, 213] Deranged inhibition of T helper cells expressing interleukin-17A (TH17 cells) [14]
Increased CA-mediated activity of Cytochrome P450 8B1 (CYP8B1)	Altered repairing ability of the intestinal mucosal barrier [39]
Altered gut metabolome [126]	Deranged BA biotransformation

Legend: CA, cholic acid; GPBAR1, G-protein-coupled BA receptor-1

Early findings documented increased levels of unconjugated BA in subjects with ulcerative colitis (UC) or Crohn’s disease (CD), as compared with healthy subjects. Changes included a decreased BA pool size in CD, but not in UC patients [31]. Nihlin et al. [32] used tauroselcholic [(selenium-75) acid] to assess BA malabsorption and BA pool loss. The authors found BA malabsorption in CD patients and this finding can explain, at least in part, the chronic diarrhea.

Zhen-Huan Yang et al. [33] investigated the relationships between BA, gut microbiota, and gut inflammation in patients with UC. The study found gut dysbiosis with decreased population of *Firmicutes*, *Butyricicoccus*, *Clostridium* XlVa, *Faecalibacterium*, and *Roseburia*, and increased pathogens such as *E. Coli*, *Proteobacteria*, *Klebsiella*, and *Streptococcus*. This deranged microbiota profile was associated in feces with decreased amounts of secondary BA concentration (LCA, DCA, glyco-conjugated GDCA, GLCA, and tauro-conjugated TLCA), and with increased concentrations of primary BA (TCA, CA, TCDA, and GCDA).

In patients with active IBD, another study showed increased rates of conjugated BA and decreased rates of secondary BA profile, as compared with controls [34]. Changes of BA profiles were associated with altered fecal microbiota, i.e., decreased ratio between *Faecalibacterium prausnitzii* and *Escherichia coli*, and with significantly decreased bacterial activities of deconjugation, transformation and de-sulphation of BA. The evidence suggests that the presence of gut dysbiosis reduces the anti-inflammatory effects promoted by secondary BA, due to their increased sulphation [34].

Decreased serum levels of BA have been documented in patients with CD, depending on altered intestinal re-absorption of BA at the level of terminal ileum. In UC patients, the level of deoxy-BA such as DCA, LCA, and conjugates was decreased in comparison to healthy and CD subjects, depending on altered colonic microbiota and, in turn, on a decreased deoxidation capacity (7α- dihydroxylation) responsible for the biotransformation of unconjugated to secondary BA [35].

A cross-sectional study measuring the plasma concentrations of 12 BA in patients with CD found decreased GCDCA, TCA, and LCA, and increased GDCA and GCA in patients, as compared with controls [36].

A recent and comprehensive review described, in IBD patients, increased fecal concentrations of CA, CDCA, conjugated BA, sulphated BA, and decreased DCA and secondary BA, as compared with healthy controls [17]. In serum, few studies reported increased GCA concentrations, and reduced LCA, GCDCA, TCDCA, TCA levels in IBD [17]. These findings can be affected by different disease type such as CD or UC, clinical status such as active/inactive disease, and location of inflammatory changes [17, 37]. Recently, however, significantly increased excretion of fecal BA was reported in patients with UC and pan-colonic disease, in a significant proportion of patients with CD affecting ileum or colon, and even in UC or CD patients with quiescent inflammation [38].

During colitis, the activation of hepatic CYP8B1, the cytochrome synthetizing CA, promotes the accumulation of intestinal CA. Consequently, CA inhibits peroxisome proliferator-activated receptor alpha (PPARα) with a decrease in fatty acid oxidation, and markedly affects the renewal of Lgr5 + intestinal stem cells. This pathway ultimately impairs the repairing ability of the gut mucosal barrier, promoting more severe colitis [39].

A longitudinal study of 1 year assessing the gut microbiome in 132 subjects found that gut dysbiosis was associated with IBD. Microbial changes included increased facultative anaerobes, decreased obligate anaerobes, and this profile occurred with decreased rates of secondary BA, i.e., LCA and DCA, and molecular disruptions in microbial transcription and metabolite pools such as short-chain fatty acids [40].

Both T helper 17 cells (Th17) expressing interleukin-17A, and regulatory T cells (Treg) play a critical role in IBD and become sensitive to changes of BA homeostasis. An imbalance between Th17 which promotes tissue inflammation and Treg which suppresses autoimmunity contribute to the onset and progression of IBD. Both gut microbiota and BA [13] can influence the production and maintenance of these immunological cells [41]. The LCA metabolite 3-oxo-LCA inhibits the differentiation of TH17 cells, while the other LCA derivative isoallo-LCA increases the differentiation of Treg cells [13]. Notably, IBD patients display a marked reduction of 3-oxoLCA, iso-LCA and the 3α-hydroxysteroid dehydrogenase (3α-HSDH) genes required for their biosynthesis. The reduced expression of TH17 cell-associated genes depends on the increased levels of these BA, which can strongly influence the onset and progression of IBD [14].

## Deranged BA signaling

BA are well-known signaling molecules interacting with nuclear and membrane-associated receptors [7, 15].

FXR is the main sensor of BA in the intestine and the liver and regulates BA synthesis by negative feedback mechanisms which also involve the intestinal secretion of the fibroblast growth factor 19 in humans [7, 15, 42, 43]. Feedbacks are deeply connected with the enterohepatic circulation of BA [44] and with the profile of gut microbiota in health and disease [1, 45]. The signaling role of BA additional receptors include the GPBAR-1 [2, 46], and the sphingosine-1-phosphate receptor 2 (S1PR2) [47, 48] in the intestine, in the liver, in the muscle and in the brown adipose tissue [7, 49], and the retinoid X receptor (RXR), the small heterodimer partner (SHP), the liver receptor homologous-1 (LRH-1), and liver X receptor (LXR) in the liver [49].

As a consequence of these inter-related pathways, altered signaling secondary to disrupted BA homeostasis may lead to multi-level dysfunction in the liver, i.e., intrahepatic cholestasis [50], liver steatosis, fibrosis, and hepatocellular carcinoma [1, 51]. At the extrahepatic level, derangement of BA homeostasis can contribute to extraintestinal cancer [51] and may affect energy expenditure [52, 53], glucose homeostasis [54], lipid homeostasis [55–58], inflammatory and immune responses [59, 60].

In the liver, FXR plays an anti-inflammatory role by reducing the availability of toxic BA especially during cholestasis [61, 62]. FXR activation inhibits monocytes/macrophages migration and tissue infiltration promoted by the chemokine monocyte chemoattractant protein-1 (MCP-1/CCL2). This step contributes to reduce liver fibrosis [62]. The use of FXR agonists leads to a reduced hepatic inflammation and fibrosis secondary to a concentration-dependent suppression of NF-κB-mediated inflammation [61]. FXR activation also stimulates anti-fibrotic gene expression in hepatic stellate cells (HSCs) through activation of FXR, induction of SHP, increased expression of peroxisomal proliferator-activated receptor γ (PPARγ) [63, 64]. Finally, in the liver, BA can have pro-inflammatory effects mediated by the intracellular assembly of the inflammasome. In this context, FXR is able to interact with the NACHT LRR and PYD domains-containing protein 3 (NLRP3) protein machinery, generating anti-inflammatory effects [65].

In the intestine, FXR has anti-inflammatory effects mainly preserving the integrity of the gut barrier [4, 7], modulating immune and inflammatory pathways by a regulation of cells involved in innate immunity [66, 67], and modulating the composition of gut microbiota [68].

In a context of deranged BA signaling, FXR- and GPBAR1-mediated immune effects can play a role through the modulatory effector functions in cells of innate immunity. In these cells, FXR activation generates a tolerogenic phenotype either at hepatic and intestinal level, with marked anti-inflammatory and anti-fibrogenic effects. However, the translational value of results from animal studies showing a role of BA as effective immune modulators in humans is still poorly documented [69].

As observed in the liver, the relationships between BA, FXR, and inflammatory pathways involving NF-κB are also active at intestinal level. DCA levels in feces can increase in response to a high-fat diet and this step is associated with increased rate of Gram-positive bacteria [70]. In the intestine, increased DCA concentration has been linked with gut inflammation and carcinogenesis. DCA-treated APC (min/ +) mice showed altered gut barrier, low-grade gut inflammation, and tumor progression [71]. DCA is able to promote colonic pro-inflammatory macrophage infiltration, pro-inflammatory cytokine production, and macrophage polarization through NF-κB/ERK/JNK signaling downstream of toll-like receptor 2 (TLR2), driving colonic inflammation [70].

FXR activation inhibits NF-κB at the intestinal level, with local anti-inflammatory effects. In animal models, FXR target gene expression (but nor mRNA expression) is decreased by inflammatory stimuli through NF-κB [66]. In addition, FXR activation decreases epithelial permeability and modulates the expression of genes involved in gut inflammation [66].

Besides FXR, the NF-κB-mediated inflammatory pathway in the intestine can be suppressed by the pregnane X receptor (PXR) [72], another nuclear receptor involved in IBD pathogenesis [66, 67, 72]. In the animal model, the administration of a PXR agonist protected wild type but not PXR-null mice from colitis induced by dextran sulphate sodium, decreasing mRNA expression of several NF-κB target genes [72]

The anti-inflammatory role of FXR is evident in *Fxr − / − *mice. These animals show a marked pro-inflammatory cytokine mRNA expression in the colon. Of note, the administration of the FXR ligand 6-ECDCA inhibits the expression of pro-inflammatory molecules in wild type but not in *Fxr − / − *animals [67].

FXR modulates the expression of several genes involved in gut permeability and inflammation, two factors involved in intestinal bacterial overgrowth [66–68]. FXR can inhibit bacterial overgrowth and mucosal injury in the ileum following bile duct ligation. This FXR-mediated effect protects the distal ileum from bacterial invasion and epithelial damage [68]. The beneficial role of FXR activation on intestinal inflammation seems to depend on FXR interaction with genes promoting antibacterial effects, i.e., genes encoding angiogenin, carbonic anhydrase 12, and inducible nitric oxide synthase, and on induction of IL-18 [68].

A study exploring the relationships between plasma BA profile and FXR/PXR activation in patients with CD found a reduced activation of target genes secondary to the deranged BA composition and, in turn, to the altered BA signaling [36]. Notably, the reduced FXR/PXR agonism can negatively affect the progression of IBD [66, 67, 72, 73].

Finally, a critical role is emerging for GPBAR1, the cell surface BA-activated receptor highly expressed in the ileum and colon [7]. The susceptibility to develop a severe colitis is significantly increased in GPBAR1(-/-) mice, due to marked alterations in the intestinal barrier [74]. On the other hand, in animal models, GPBAR1 agonists prevent gut inflammation [75]. A recent study in patients with CD demonstrated that GPBAR1 can modulate, in the colon, the expression of ACE2 [76], a receptor involved in intestinal inflammatory processes [77] and able to attenuate intestinal inflammation [76].

## Deranged BA–microbiota axis

The gut barrier is an anatomical and functional structure at the border between external environment, i.e., the gut lumen and the host body [4]. The integrity of the barrier depends on the dynamic interaction between several factors: gut microbiota, luminal content of nutrients, mucin, gastrointestinal motility, and secretions, i.e., gastric acid, bile, pancreatic juice, intestinal cells, i.e., enterocytes, Paneth cells, Goblet cells with their tight junctions. Essential components of the gut barrier include also immune-modulating components such as antimicrobial peptides, i.e., microbial- [MAMPs] and pathogen-[PAMPs] associated molecular patterns, toll-like receptors [TLRs], B/T lymphocytes, and cells composing the gut-vascular barrier, i.e., endothelium associated with pericytes and enteric glial cells with specific tight junction and adherens junctions.

As part of the gut barrier machinery, the microbiota and BA have a critical role in maintaining the integrity of the intestinal barrier due to the close bidirectional crosstalk [49, 78–81] and potential influence on the onset and progression of chronic intestinal inflammation [82]. Of note, a dysfunction of the gut barrier can precede and predict the development of IBD by years [83, 84]. Table 2 lists the main mechanisms linking gut dysbiosis with the pathogenesis of IBD, all pointing to a critical involvement in both local inflammation and altered intestinal barrier.

**Table 2:** Main mechanisms linking gut dysbiosis with the pathogenesis of IBD

Microbial invasion of the gut mucosa in IBD patients (both CD and UC) [214]
Altered expression of host genes [40, 127, 128]
Epigenetic upregulation of colitis-associated gene expression (AP1, FOSL2, FRA1) [215]
Decreased production of bacterial metabolites (mainly secondary BA, SCFAs [104, 105, 216], Acyl-homoserine lactones (AHL) [217]
Increased production of lipopolysaccharides (LPSs) [95]
Deficient tryptophan metabolism [106]
Altered production and maintenance of T helper 17 cells expressing interleukin-17A (Th17), and regulatory T cells (Treg) [13, 41, 172]
Negative effects on innate lymphoid cells (ILCs) (activation of ILC3 and dendritic cells, differentiation from ILC3 toward ILC1 [218, 219], increased production of IL-22, IL-17, interferon-γ [220])

As compared with healthy individuals, IBD patients show reduced bacterial abundance and diversity [82, 85], with a decrease of *Firmicutes* and *Bacteroidetes,* and increased *Proteobacteria* and *Enterobacteriaceae* [33, 86–89]. Reduced bacterial diversity has been described in both inflamed and non-inflamed colon sites in patients with IBD, although inflamed sites seem enriched with specific bacterial species i.e., *Cloacibacterium* and *Tissierellaceae*, as compared with non-inflamed tissues [90]. The relative abundance of gut microbes also changes with the activity of IBD, and a lower abundances of *Clostridium coccoides, Clostridium leptum, F. prausnitzii,* and *Bifidobacterium* has been linked with periods of disease remission [91]. Despite the association between gut dysbiosis and IBD has been well documented, the causal role of altered gut microbiota in the determination of chronic intestinal inflammation is still under debate. The shift of microbiome in IBD patients may represent a microbial response secondary to local inflammatory changes, rather than having a causal role [85, 92]. Nevertheless, several gut inflammatory pathways can be activated by unbalance between harmful and beneficial gut microbes [93, 94].This condition occurs during upregulation of pathogenic bacteria species, i.e., *Enterobacteriaceae* [95, 96], *Clostridium difficile* [97], and decreased abundances of beneficial bacteria species, i.e., *Clostridium* clusters IV and XIVa, *Faecalibacterium prausnitzii*, *Eubacterium* [98, 99]. This unbalance may also lead to increased production of pro-inflammatory lipopolysaccharides (LPSs) and their filtration across the altered gut barrier unable to maintain a selective normal permeability [95]. In line with this evidence, specific bacterial species, (i.e., *Lactobacillus*, and *Faecalibacterium* within *Firmicutes*; *Bifidobacterium* within *Actinobacteria*) [92] can have a beneficial role in IBD patients.

Of note, the reduced microbial abundance in IBD patients involves bacteria like *Bacteroides*, *Clostridium, Lactobacillus*, *Bifidobacterium*, and *Listeria* carrying bile salt hydrolase (BSH), the enzyme involved in the biotransformation of conjugated into unconjugated BA [100, 101], and microbes (mainly *Bacteroides*, *Clostridium*, *Eubacterium*, and *Lactobacillus*) responsible for the 7α-dehydroxylation of unconjugated BA and, therefore, for their bio-transformation to secondary BA [102].

As shown in an animal models, the cecal concentrations of UDCA and LCA, its primary metabolite, were protective against the disruption of epithelial permeability and colonic inflammation, inhibiting colonic epithelial caspase-3 cleavage and epithelial apoptosis [103].

In a group of patients with UC, the reduced diversity of gut microbiota as compared with healthy controls was in line with decreased microbes such as *Firmicutes, Clostridium IV, Butyricicoccus, Clostridium XlVa, Faecalibacterium,* and *Roseburia*, and enrichment in *Proteobacteria*, *Escherichia*, *Enterococcus*, *Klebsiella*, and *Streptococcus*. These changes caused a significant decrease of secondary BA, with increased primary BA, altered GPBAR1 expression, and increased production of pro-inflammatory cytokines [33].

As previously mentioned, the link between gut dysbiosis and altered profile of gut BA can reduce the FXR/PXR agonism, while promoting the IBD progression through altered BA signaling functions [66, 67, 72, 73]. The altered intestinal profile of BA secondary to dysbiosis can affect the intestinal permeability, together with the dysregulation of bacterial metabolites usually contributing to the maintenance of the integrity of gut barrier, as short-chain fatty acids (SCFA) like butyrate, acetate, and propionate [104, 105]. In a mouse model of autism spectrum disorders, a reduction in the relative abundance of *Bifidobacterium* and *Blautia*, bile-metabolizing species, was linked in the intestine with deficient BA and tryptophan metabolism and with increased intestinal macromolecular permeability [106]. Cytotoxic effects of elevated concentration of BA on the intestinal epithelium have been observed in cells, animals, and humans, and are able to affect the integrity of the gut barrier [107–109]. These effects are mediated by different inflammatory and apoptotic molecules as phospholipase A2 (PLA2)- cyclooxygenase (COX)-protein kinase C (PKC), extracellular signal-regulated kinase 1 (ERK1), p38 mitogen-activated protein kinase (p38 MAPK), and phosphatidylinositol 3-kinase (PI3K), which can be activated by altered intestinal BA profile [110–117].

In vitro models of gut barrier based on monolayers of human intestinal Caco-2 cells contributed to document the negative, cytotoxic effects of hydrophobic BA (mainly unconjugated BA), possibly leading to increased gut permeability and inflammation. In this model, CA decreases the transepithelial electrical resistance (TEER) and increases intracellular ROS generation. These effects seem to be mediated by the activation of signaling pathway involving PLA2, COX, PKC ERK1/2, PI3K, p38 MAPK, MLCK, NADH dehydrogenase, and XO (xanthine oxidase) [118]. In the same cellular model, CA, DCA, and CDCA, but not UDCA, decreases TEER and increase paracellular permeability [119]. Furthermore, CDCA or DCA promoted a ligand-independent activation of the epithelial growth factor receptor (EGFR), which correlates with increased paracellular permeability via occludin dephosphorylation and cytoskeletal rearrangement at the tight junctions [119].

In animal models (mice) of colitis, increased intestinal permeability at the level of the colon was linked with decreased proportion of UDCA, increased DCA, and increased jejunal FXR expression [120, 121]. Furthermore, mice with colitis induced by dextran sodium sulphate (DSS) show increased fecal BA hydrophobicity. Notably, the severity of symptoms correlated positively with fecal BA hydrophobicity and fecal DCA concentration [122].

Mice fed a choline-deficient, l-amino acid- deficient, high-fat diet showed reduced concentrations of conjugated BA, which was paralleled by increased gut permeability. In vitro, conjugated BA protected gut epithelial monolayers from the damage induced by unconjugated BA through micelle formation [123].

In mice with DSS-induced colitis, gut inflammation worsened after administration of a ketogenic diet, which induced an upregulation of serum and colon inflammatory cytokines and chemokines (IL-1α, IL-6, TNF-α, IL-17, GM-CSF and IL-10), increased gut permeability, and decreased the expression of intestinal-epithelial-barrier-associated genes. These changes were linked with significant variations in bacterial abundance, i.e., increased pathogenic taxa as *Proteobacteria*, *Enterobacteriaceae*, *Helicobacter*, *Escherichia-Shigella*; reduced beneficial taxa as *Erysipelotrichaceae*, and with altered concentration of microbial metabolites, including BA (i.e., increased TCDCA, CA, GCA) [124].

Impaired BA homeostasis can significantly affect the modulatory role of BA on the proliferation of epithelial cells [2, 3], gene expression [5, 6], and epigenetic mechanisms [8, 9], including the interactions between microbial and host genes [125] and the gut metabolome, the molecular interface between host and microbiota [126]. In IBD patients, variations in the relative abundance of mucosa-adherent microorganisms are able to modulate the expression of several host genes [40, 127, 128], and an altered BA homeostasis seems to have a critical role in this process [129].

A study on colonic biopsies from patients with primary sclerosing cholangitis (PSC), who frequently have colitis, UC patients and healthy controls reported different microbiota profiles and significantly different colonic transcriptome, with 939 genes sharing differential gene expression in patients (both UC and PSC), as compared with controls. In patients, imputed pathways were linked with upregulation of immune response and microbial defense, and BA signaling pathways were upregulated in PSC-IBD, as compared with UC [129].

Finally, a study on endoscopic mucosal biopsies (ileum and colon) from IBD patients documented a deficient microbial gene pathway involved in the biosynthesis of secondary BA in inflamed terminal ileum. In samples from non-inflamed colon, the relative abundance of BA-inducible microbial genes directly correlated with the expression, in the host, of Angiopoietin-like 4 (*Angptl4*) [125], a gene able to attenuate colonic inflammation in animal models [130]. The correlation between BA-inducible microbial genes and *Angptl4* gene expression disappeared with inflammation [125].

## Potential therapeutic implications

The available evidence suggests that there is a link between IBD and BA homeostasis, and that there is a room for potential therapeutic approaches that can modify the clinical course of disease. Most relevant approaches include BA therapy, gut microbiota modulation, and use of potent FXR agonists.

### Therapy with BA

Therapeutic approaches for liver diseases have used hydrophilic BA, i.e., the “tertiary” UDCA acid, the conjugated tauro-UDCA, and, more recently, nor-UDCA [131]. This strategy decreases the hydrophobicity of the BA pool and the cytotoxic effect which occurs at the level of enterocytes [132].

In an animal model of CD, the administration of UDCA was beneficial through positive effects on the intestinal barrier and by reducing the oxidative stress [133]. In the animal models of IBD, the intraperitoneal administration of UDCA and LCA had protective effects against increased epithelial permeability and colonic inflammation. The mechanism included the inhibition of epithelial apoptosis [103] and cytoprotective and anti-inflammatory effects [134].

The beneficial effects of tertiary BA also depend, at least in part, on changes in gut microbiota secondary to the mutated intraluminal BA concentration. In mice UDCA, TUDCA, GUDCA restored the *Firmicutes* to *Bacteroidetes* ratio after a colitis-induced dysbiosis, prevented the loss of *Clostridium cluster* XIVa, and increased the abundance of protective species (in particular *Akkermansia muciniphila*) [135].

Looking at the effect of BA therapy in IBD, available results in humans are scarce and need further confirmation. Preliminary evidence in UC patients found better therapeutic effects, i.e., reduced Mayo and IBDQ scores when UDCA 200 mg b.i.d. was added to mesalamine. Of note, the combined treatment was also able to modulate the gut microbiota by increased *Firmicutes* and reduced *Proteobacteria*, as compared with subjects on mesalamine alone [136].

To counteract the altered BA balance documented in IBD patients, a displacement therapy should be aimed to inhibit the synthesis of primary BA or to increase the fecal elimination of toxic BA through BA binders, as cholestyramine. In an animal model of IBD, cholestyramine attenuates intestinal ulceration [137]. In subjects with collagenous colitis, adding cholestyramine (4 g/day) to mesalamine increases the rate of beneficial therapeutic response (100%, as compared with 73% in mesalamine alone) [138]. The use of cholestyramine is indicated to counteract chronic diarrhea linked with BA malabsorption in CD [32]. In patients with IBD linked with primary sclerosing cholangitis and receiving optimized anti-TNF therapy for IBD, the use of cholestyramine induced a rapid and sustained drop in fecal calprotectin levels [139].

### Therapy with probiotics and prebiotics

According to WHO and FAO, probiotics are “live microorganisms when administered in adequate amounts confer a health benefit on the host”. The administration of probiotics (mainly *Lactobacillus* [140], *Bifidobacterium* [141, 142], *S. boulardii* [143, 144]*, L. rhamnosus GG* [145–148], *L. johnsonii LA1* [149, 150], *E. faecium* [146]*, VSL#3* [151, 152]*, E. Nissle 1917* [153–155]) can have beneficial effects in IBD patients by acting on the microbiota/BA axis. The therapeutic effects of probiotics likely involve improved gut barrier function and the recovery of physiological gut microbiota involved in the bio-transformation and homeostasis of BA, and ultimately modulating the profile of the luminal pool of BA [156].

In animal models and in humans, additional therapeutic effects of probiotics (mainly *Lactobacillus plantarum CCFM8661, Lactobacillus reuteri NCIMB 30242, VSL#3*) involve the activation of the fibroblast growth factor (FGF)19 and 15 [157–159] and, in turn, enhanced synthesis and excretion of BA [15].

Results of controlled trials using probiotics, however, are controversial with few studies reporting no effects on relieving relapse [143, 150, 160] and uncertain beneficial effects [153, 161]. A meta-analysis exploring ten randomized controlled trials found that probiotics can induce remission during the active period of UC, but have no significant effects in maintaining CD and UC remission [162]. Another recent systematic review on the use of probiotics in IBD patients reported no clear beneficial effects in CD patients, but positive effects in inducing remission in patients with active UC [163].

In a recent study in IBD patients, the probiotic strain *Bacillus clausii* UBBC-07 positively modulated the gut microbiota and cytokine secretion, and was associated with a significant decrease of symptoms [164].

*Akkermansia muciniphila* represents 1–4% of gut microbiota in healthy humans [165]. IBD patients show decreased rates of *A. muciniphila* [165, 166] and, in the mice models of colitis, the administration of *A. muciniphila* improves intestinal permeability [167], decreases colon inflammation and the expression of pro-inflammatory cytokines (TNF-α, IFN-γ) [168]. In mice, the administration of protein components of the outer membrane protein from *A. muciniphila* protects from the development of colitis [169]. *A. muciniphila* can also play a role in the modulation of immune responses mediated by the Toll-like receptor 4 (TLR4), a sensor of gut microbiota alterations sensible to the intestinal concentration of pathogen-associated molecular patterns (PAMPs) and damage-associated molecular patterns (DAMPs) [170, 171]. A recent study in TLR4-/- mice reported a protective role of TLR4 against the development of intestinal inflammation, linked with the relative abundance of *A. muciniphila* and the proportion of suppressive RORγt + Treg cells [172]. The close crosstalk between microbiota and BA was disclosed by an experimental model of dextran sodium sulfate-induced colitis in mice, since the administration of UDCA decreased the inflammatory changes and increased the abundance of *A. muciniphila* [135]. In another animal model of early obesity and non-alcoholic fatty liver disease, the administration of *A. muciniphila* was associated with increased plasma levels of unconjugated, hydrophilic BA and with increased expression of hepatic genes involved in BA synthesis and transport, pointing to a critical role of *A. muciniphila* in the modulation of BA signaling [173].

Prebiotics are substrates selectively employed by gut microbes, providing beneficial effects as the development of probiotics (including *Ruminococcaceae, Lachnospiraceae,* and *Bifidobacterium*) and the formation of metabolites as SCFAs and BA [174–177].

The most common prebiotics employed in IBD are lactulose [178], fructo-oligosaccharide (FOS) [179], germinated barley foodstuff [180, 181], ispaghula husk [182], Plantago ovata seeds [183], and inulin [184, 185]. However, the effects of chronic supplementation with these prebiotics on BA homeostasis in IBD patients are still scarcely explored and lead to uncertain results.

In humans, chronic ingestion of lactulose seems to be able to increase *Bifidobacteria* but not to significantly change fecal BA [186, 187]. Nevertheless, in a previous study, 12 weeks of lactulose 60 g/day decreased secondary BA absorption, decreasing the DCA pool size, with a rise in primary BA [188].

In healthy subjects, it has been reported that long-term FOS administration is able to decrease fecal DCA [189]. A previous evidence, however, was unable to demonstrate significant changes in fecal BA concentration [190].

In an experimental model of colitis, mice receiving germinated barley foodstuff showed a reduced epithelial inflammatory response, paralleled by increased butyrate production and lower BA concentration, as compared with control animals [191]. This dietary fiber is able, in vitro, to strongly adsorb hydrophobic bile salts [192]. However, studies exploring the effects of germinated barley foodstuff on BA homeostasis in humans are still lacking.

The effects of long-term (8 weeks) supplementation with ispaghula husk on the fecal output of BA have been explored in healthy adult subjects, showing a significant decrease of fecal LCA and iso-LCA and the weighted ratio of LCA to DCA, pointing to a reduction of the hydrophobicity of the BA pool [193].

Plantago ovata seeds had no effect on fecal BA excretion in a small group of normal subjects [194]. In guinea pigs, however, the husks from Plantago ovata significantly increased fecal BA, affecting BA absorption [195].

Finally, in animals, inulin increases the fecal concentration of DCA and LCA [177] and changes the composition of gut microbiota and the levels of related metabolites, as BA [185]. The effects of inulin decrease with deletion of FXR, and modulate the pathogenic mechanisms involved in chronic gut inflammation [185]. In patients with an ileal pouch-anal anastomosis, the administration of 24 g of inulin during 3 weeks decreased the numbers of *Bacteroides fragi*lis and reduced the fecal concentrations of secondary BA, with beneficial effects on the mucosal inflammation in the ileal reservoir[196].

### Agonists of BA receptors

Studies in animal models documented beneficial effects from FXR activation by specific agonists as INT-747 [11], fexaramine [197], and GW4064 [198] documented by prevention of colitis, anti-inflammatory effects, restored BA homeostasis, and gut microbiota modulation. In a mouse model of colitis, INT-747 alleviated colon inflammation downregulating pro-inflammatory cytokines and preserving gut barrier function [11, 67]. In mice with DCA-induced intestinal damage, the administration of fexaramine decreased the injury, increased the abundance of SCFA-producing bacteria, and normalized BA homeostasis through beneficial effects of the FXR/FGF15 axis [197]. The administration of GW4064 generated favorable effects in an animal model of ileum injury induced by lipopolysaccharides decreasing tight junction dysfunction, macrophage infiltration, inflammatory pathways, and mitochondrial dysfunction with FXR-dependent mechanisms [198]. However, results from another in vitro study on colonic epithelial restitution and wound healing in T_84_ cell monolayers documented an harmful inhibition of wound closure by GW4064, with a downregulation of CFTR gene expression [199].

## Conclusion and future perspectives

**Fig. 2:**
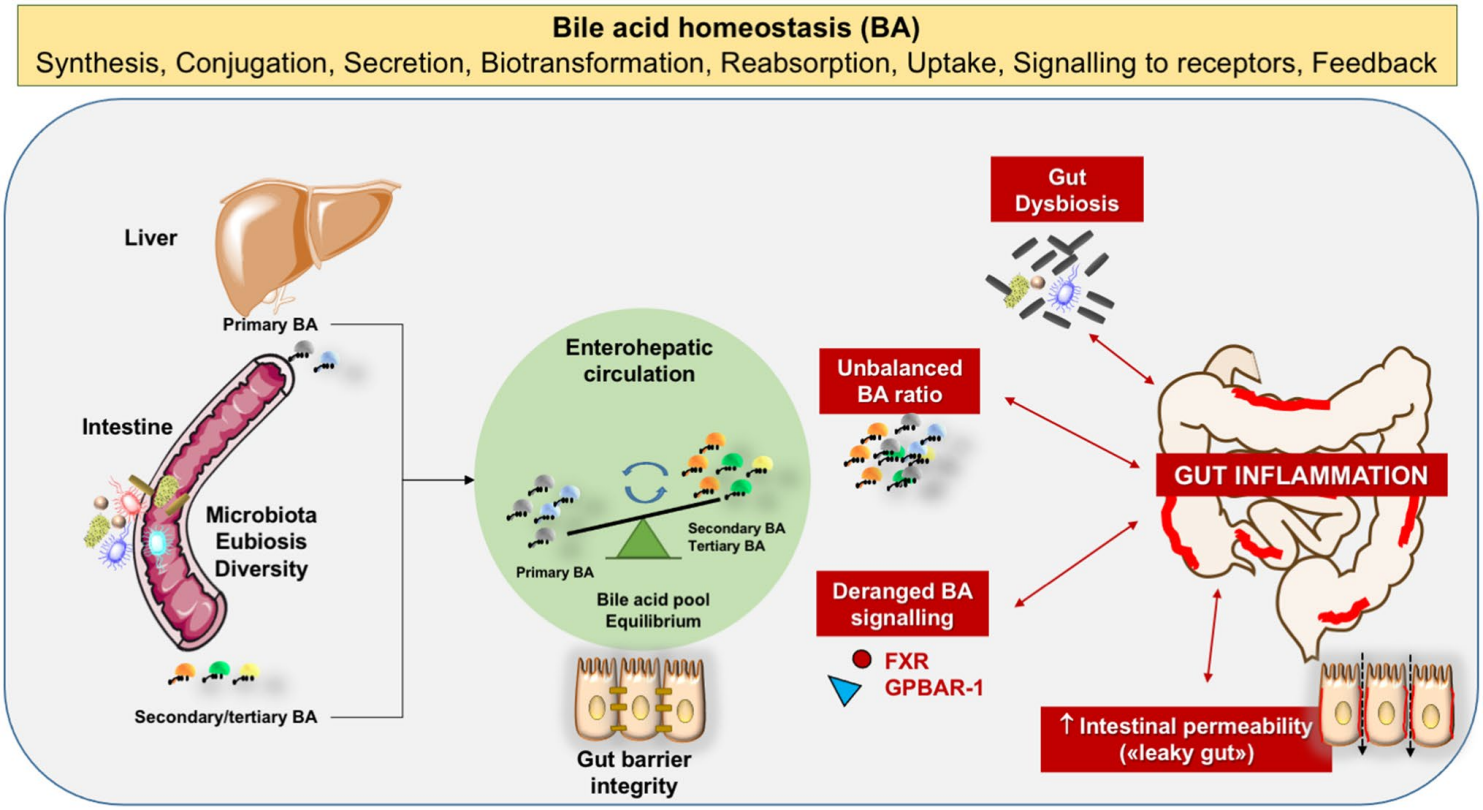
Summary of major mechanisms involved in the interaction of bile acids with gut inflammation in the pathogenesis of inflammatory bowel disease. Disturbed BA homeostasis can activate pro-inflammatory pathways in the gut, and inflammatory bowel disease (IBD) can, in turn, induce qualitative and/or quantitative changes in the BA pool in response to gut dysbiosis, leading to impaired mucosal barrier repair due to chronic inflammation. Legend: *BA* bile acids, *FXR* farnesoid X receptor, *GPBAR1* G protein -coupled bile acid receptor-1

A dynamic crosstalk exists between BA homeostasis which includes signaling effects on nuclear and membrane receptors, gut microbiota, and maintenance of gut barrier integrity. These critical factors can become actors in the onset and progression of chronic intestinal inflammatory diseases (Fig. 2). More studies must identify key aspects lacking the full translational value. In particular:

1. The potential links between gut dysbiosis, BA homeostasis, and IBD pathogenesis, point to novel therapeutic strategies. The translational value of available animal and experimental studies, however, must be confirmed in clinical trials considering the role of confounders such as age, dietary habits, lifestyle, ethnicity, drugs, possible chronic ingestion of toxic chemicals with diet, altered metabolic homeostasis, and comorbidities. In humans, the combination of these factors limits the ultimate identification of the causal role of gut dysbiosis in the onset and progression of chronic intestinal inflammation. Well-designed, accurate, and prospective studies are needed with respect to gene–environment interactions, and epigenetic mechanisms. oth artificial intelligence and multiomics can provide additional information in this respect [200, 201]. Starting from machine learning models [202, 203], these techniques will likely contribute to the advancement in the knowledge of the pathogenic mechanisms linking BA, gut microbiota, and gut inflammation. Results will facilitate disease management and can pave the way to primary prevention measures.
2. Animal studies reveal beneficial interplays between gut microbiota variations and anti-inflammatory effects of secondary and tertiary BA. However, the effective value of microbiota transplantation and/or of BA therapy in the management of humans with IBD is still uncertain. Although promising results derive from experimental studies with hydrophilic BA, large randomized, controlled trials targeting the role of BA therapy are still needed. Further studies are also expected to verify the possible convenience, in humans, of BA displacement therapy using BA binders as cholestyramine.
3. BA are signaling molecules for FXR and GPBAR-1. The interactions between BA and membrane/nuclear receptors can generate anti-inflammatory and immune-modulating effects at the intestinal level, mainly acting on cells involved in innate immunity. Preclinical studies indicate that external manipulation of the BA receptors (mainly FXR) with specific agonists can have positive effects in terms of both clinical remission during active periods and maintenance of remissions. To date, however, results in humans are scarce and need further confirmation, also in terms of combination with standard treatments. Although a number of clinical trials are on the way using FXR agonists in chronic liver diseases and in several metabolic disorders [15], no evidence exists on the use of these agents in humans with IBD. We need caution when considering the potential negative effects on healing of the inflamed colon and on expression of genes involved in the maintenance of gut barrier [199].
4. The precise therapeutical efficacy of prebiotics and/or probiotics in patients with IBD requires additional validation and well-designed randomized controlled trials. Promising results derive from the supplementation with some probiotics (mainly *Lactobacilli*, *Bifidobacteria*, and *Akkermansia muciniphila*) and prebiotics (mainly germinated barley foodstuff and inulin). Long-term effects of such therapeutic approaches are also uncertain.
5. We need to clarify if combined multifaceted approaches (including lifestyle changes, environmental exposures, and innovative drugs) aimed at restoring BA homeostasis and gut dysbiosis, do have additional value in the short and in the long term as compared with conventional drug treatment.

## Data Availability

Not applicable.
